# Impedance Problems and Their Causes—A Single-Center Analysis of 601 Patients with De Novo Deep Brain Stimulation

**DOI:** 10.3390/jcm15020683

**Published:** 2026-01-14

**Authors:** Thomas Fortmann, Samer Zawy Alsofy, Antonio Santacroce, Makoto Nakamura, Christian Ewelt, Ralph Lehrke

**Affiliations:** 1 Department of Stereotactic Neurosurgery, St. Barbara Hospital Hamm, 59073 Hamm, Germany; rlehrke@barbaraklinik.de; 2 Faculty of Medicine, University of Witten/Herdecke, 58455 Witten/Herdecke, Germany; szawyalsofy@barbaraklinik.de (S.Z.A.); antonio.santacroce@erc-munich.com (A.S.); nakamuram@kliniken-koeln.de (M.N.); 3 Department of Neurosurgery, St. Barbara Hospital Hamm, 59073 Hamm, Germany; cewelt@barbaraklinik.de; 4 European Radiosurgery Center Munich, 81377 Munich, Germany

**Keywords:** deep brain stimulation, impedance, revision surgery

## Abstract

**Background/Objectives**: Patients with deep brain stimulation (DBS) require regular follow-up. When a sudden loss of therapeutic effect occurs, impedance abnormalities are often the underlying cause. If reprogramming cannot restore clinical benefit, revision surgery may be necessary to replace defective hardware. Since all three major manufacturers are used at our center, we analyzed our patient cohort to determine the incidence and causes of impedance abnormalities. **Methods**: All 601 patients who underwent de novo DBS implantation in Hamm between 2009 and 2025 were evaluated for impedance abnormalities. In cases requiring revision surgery, the specific cause was identified. The manufacturer, electrodes, and contacts involved were systematically analyzed. **Results**: A total of 25 of 601 patients required revision surgery. Revision rates were 2.67% in patients with Parkinson’s disease, 6.19% in those with a tremor, and 5.71% in those with dystonia. Across manufacturers, 7.6% of patients with a Medtronic system required revision surgery, compared with 3.4% of patients with an Abbott system and no patients with a Boston Scientific system. The primary causes of revision were electrode-related problems (19/25), followed by extension defects (6/25), connector issues (4/25), and, in one case, a generator defect (1/25). **Conclusions**: Only 4.16% of patients required revision surgery due to impedance abnormalities. Patients with a tremor and non-segmented electrodes showed a higher incidence than those with Parkinson’s disease or dystonia. Predominantly older Medtronic systems had the highest revision rate, whereas no Boston Scientific systems required revision. In most cases, the electrodes were the primary source of impedance abnormalities. A total of 52% of revisions were performed within two years and 92% were performed within six years of implantation.

## 1. Introduction

Deep brain stimulation (DBS) is an established and effective treatment modality for Parkinson’s disease, essential tremor, dystonia, obsessive–compulsive disorder, and several other neurological conditions. During implantation, system impedance is intermittently measured to verify correct connections and proper functioning of the implanted electrodes, extensions, and the implantable pulse generator (IPG) [1].

Elevated impedance values observed intraoperatively are occasionally attributable to intracranial air bubbles surrounding electrode contacts [2,3,4]. In most cases, impedance levels gradually decrease over the course of the procedure. When uncertainty arises, postoperative computed tomography (CT) can confirm the presence of air bubbles around the affected contacts.

Routine impedance measurements are recommended at every follow-up visit. In a cohort of 1764 leads, Wong et al. demonstrated a progressive decline in impedance levels over the first six months following implantation. Electrodes placed within the subthalamic nucleus (STN) for Parkinson’s disease have been shown to exhibit impedance fluctuations for up to 84 months postoperatively [5].

Several reports have described a sudden loss of therapeutic efficacy in DBS therapy associated with impedance abnormalities, electrode or extension fractures, or IPG malfunction [6,7,8,9,10]. Twiddler’s syndrome, characterized by rotation of the IPG along its vertical axis—either inadvertently or through patient manipulation—can result in coiling of the extensions and traction on the leads, ultimately leading to conductor breakage [11,12]. Although rare, only 23 cases had been reported in the literature as of 2022.

Reported rates of impedance-related adverse events vary widely, ranging from 2.8% to 55% [13,14,15,16]. Over time, both high-impedance states and short circuits may occur, with or without corresponding clinical manifestations. In the majority of cases, reprogramming of the IPG restores clinical benefit [17,18]. When complications are suspected, impedance testing combined with radiographic evaluation of the entire system is essential for accurate diagnosis [19].

Between 2009 and 2025, a total of 601 de novo DBS implantations were performed at our center, utilizing devices from all major manufacturers currently available. The objective of the present study is to analyze DBS systems to determine whether specific manufacturers exhibit a higher susceptibility to impedance-related complications. Furthermore, we systematically recorded impedance issues to evaluate their temporal and anatomical distribution, management strategies, and the necessity of surgical revision. Our hypothesis is that the number of impedance problems does not differ between the three major manufacturers. The aim of this study is to analyze our patient cohort to identify risk factors. To our knowledge, no prior comparative analysis addressing these aspects in all three manufacturers has been published.

## 2. Materials and Methods

All patient records of individuals who underwent de novo deep brain stimulation (DBS) at the Stereotactic Department of St. Barbara Hospital in Hamm were retrospectively analyzed. Between January 2009 and March 2025, 601 patient records were identified and reviewed. All patients were referred by neurologists according to the German guidelines after a failure of medical treatment success. After implantation, the patients were regularly seen in our outpatient department in fixed intervals—3, 6 and 12 months after surgery and then at least yearly or upon troubleshooting. Every time, the IPG was checked, including the impedances. When impedance abnormalities occurred, reprogramming was tried. If it was not successful, X-rays were taken from the complete system. If no obvious break was seen, the system was explored surgically. Connectors and the IPG were freed and electrodes and extensions were examined for impedance problems. Defective hardware components were replaced. The impedance problems were classified according to whether they resulted in surgical revision or were just documented during follow-up. All patients that were treated by surgical revision had a reoccurrence of clinical symptoms, which were successfully treated by DBS, and could not be reprogrammed.

For each patient, the implanted electrodes, extensions, and pulse generators were recorded. The causes of impedance abnormalities and the specific contacts involved were systematically evaluated. The cohort included all commonly available DBS systems from Medtronic, Abbott (formerly St. Jude Medical), and Boston Scientific.

All procedures were performed by the same surgical team. The senior author had 13 years of DBS experience at a high-volume center before founding the Hamm program in 2009. From 2009 to 2018, one attending neurosurgeon supported him; since 2018, the first author has served in this role.

For analysis, the Chi-square test and the Chi-square contribution was estimated using Excel from Microsoft Office 365. All tables and figures were created with Excel.

## 3. Results

First, we provide an overview of our patient cohort (Table 1). The majority of patients were Caucasian (97%), with a slight majority of men amongst our patients (59%). The median age was 62 years, ranging from 15 to 85 years. The treated diseases were Parkinson’s disease, in more than half our patients, followed by tremor and dystonia.

### 3.1. Patients and Hardware

As in many centers, the most common indication for DBS implantation was Parkinson’s disease (*n* = 337), followed by tremor disorders (*n* = 194) and dystonia (*n* = 70).

**Figure 1:**
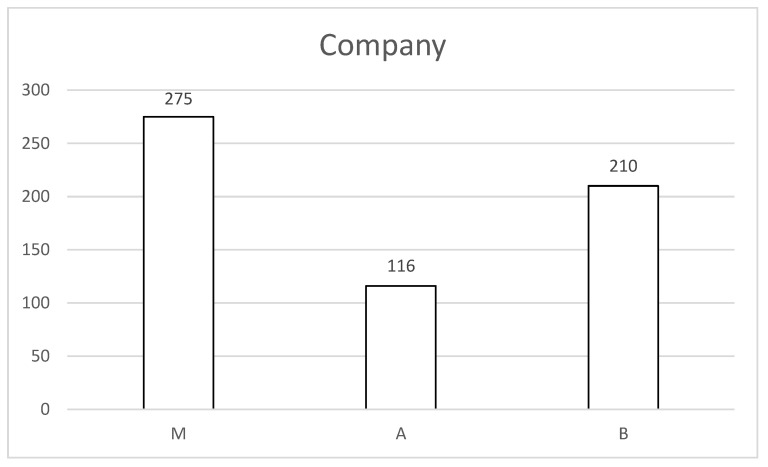
De novo implantations in relation to the supplier used. M is Medtronic, A is Abbott (formerly St. Jude Medical) and B is Boston Scientific.

When DBS became an established treatment option in Germany in the 1990s, Medtronic was the first company to provide implantable DBS systems. St. Jude Medical (now Abbott) entered the DBS market in 2009, followed by Boston Scientific in 2012. In our center, 275 patients received a Medtronic DBS system, 116 received an Abbott system, and 210 were implanted with a Boston Scientific system (Figure 1).

**Figure 2:**
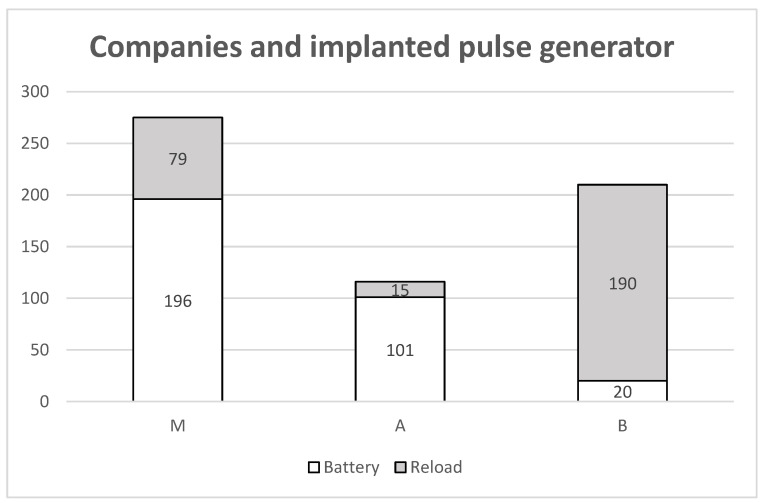
Overview of companies and the implanted pulse generator. From Medtronic and Abbott, mainly batteries were implanted; from Boston Scientific, mainly reload batteries were implanted.

Because all manufacturers offer both non-rechargeable and rechargeable implantable pulse generators, we analyzed the distribution of battery types used in our cohort. For Medtronic, 196 non-rechargeable and 79 rechargeable batteries were implanted; for Abbott, 101 non-rechargeable and 15 rechargeable batteries were used. In contrast, Boston Scientific systems included 20 non-rechargeable and 190 rechargeable batteries (Figure 2). Notably, when Boston Scientific entered the market in 2012, only rechargeable generators were available initially; non-rechargeable models were introduced later.

**Figure 3:**
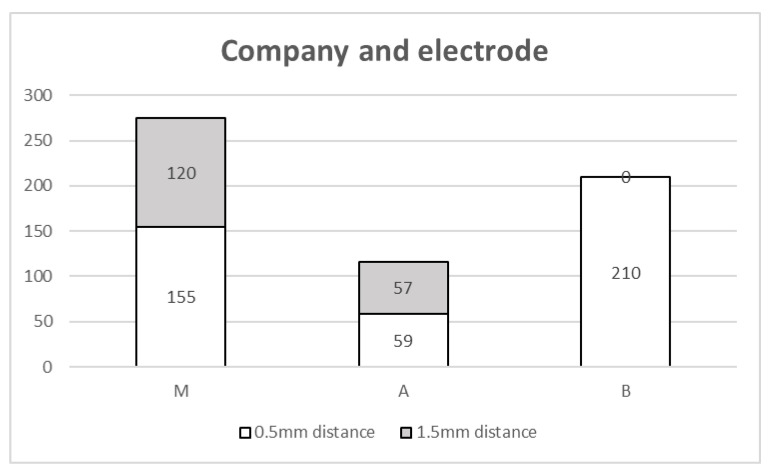
Overview of companies and the implanted electrodes. From Medtronic and Abbott, narrow spacing and wide spacing were used almost equally. Boston Scientific only offers narrow spacing.

Medtronic and Abbott offer DBS leads with both wide and narrow contact spacing, whereas Boston Scientific provides only narrow-spacing leads. In our center, wide-spacing electrodes were preferentially used for tremor cases when Medtronic or Abbott systems were implanted. Consequently, 120 Medtronic recipients received wide-spacing electrodes and 155 received narrow-spacing electrodes. Among Abbott recipients, 57 patients received wide-spacing and 59 received narrow-spacing electrodes. All 210 Boston Scientific recipients were implanted with narrow-spacing electrodes (Figure 3).

### 3.2. Impedance Problems

#### 3.2.1. Impedance Problems Overview

**Figure 4:**
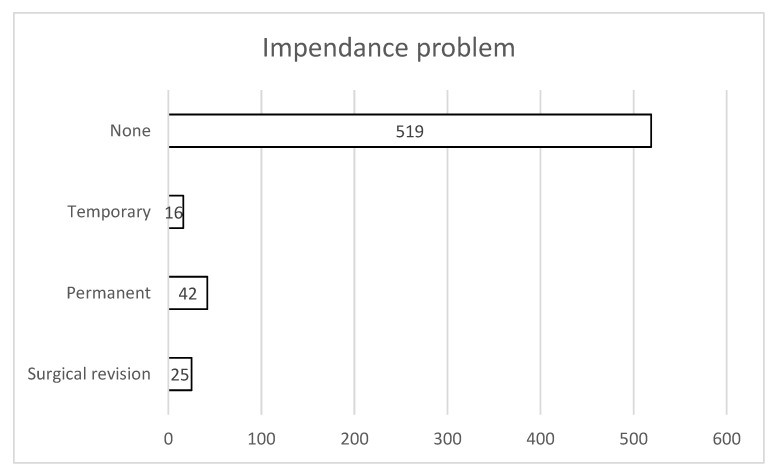
Of all implanted patients, 519 had no problems with their impedances. A total of 16 patients had temporary problems that disappeared during the follow up, 42 had permanent problems, and an additional 25 patients required surgical revision of the implanted system.

A total of 519 patients (86.4%) exhibited no impedance abnormalities at any time during follow-up. Sixteen patients (2.7%) experienced a temporary impedance issue, whereas forty-two patients (6.9%) had a persistent impedance abnormality. Twenty-five patients (4.2%) required revision surgery to correct an impedance problem (Figure 4). Of all patients with impedance abnormalities (*n* = 83), 33.7% (*n* = 25) needed surgical revision.

Among the 25 patients who required revision surgery, 9 had Parkinson’s disease (36%), 12 had a tremor (48%), and 4 had dystonia (16%) as their primary diagnosis. Compared to the percentage of patients with PD (56.07%), ET (32.28%) and D (11.65%), the Chi-square test is highly significant. For PD, the value is lower than expected and for ET it is higher (Table 2).

**Figure 5:**
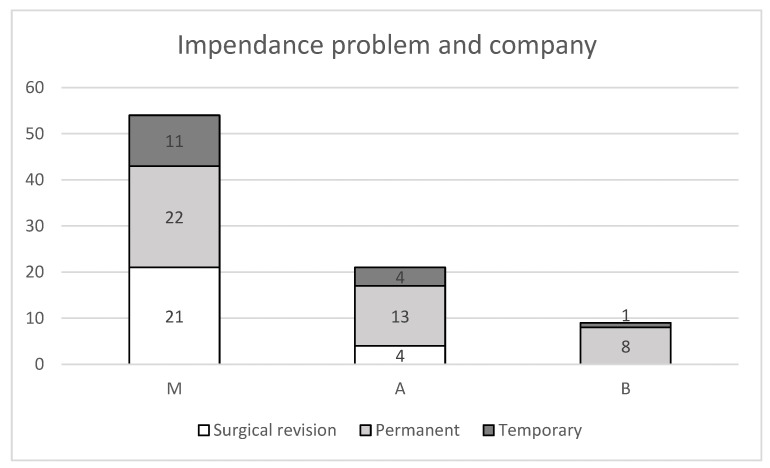
Overview of all patients with impedance problems, split up regarding the manufacturer and their severity. A total of 54 patients had impedance problems with Medtronic systems, 21 with Abbott systems and 9 with Boston Scientific systems.

In the Medtronic cohort, 53 patients experienced an impedance abnormality (11 temporary, 22 permanent, and 21 requiring revision). In the Abbott cohort, 21 patients had impedance problems (4 temporary, 13 permanent, and 4 requiring revision). In the Boston Scientific cohort, nine patients exhibited impedance abnormalities (one temporary and eight permanent); no revisions were required in this group (Figure 5).

#### 3.2.2. Impedance Problems and Surgical Revision

Among patients who required revision surgery, 7.6% of those with a Medtronic system underwent revision, compared with 3.4% of those with an Abbott system and none of the patients with a Boston Scientific system.

When a revision was performed, the electrodes were most often the source of the impedance problem and had to be renewed 23 times. Extensions were the cause 11 times; one St. Jude Libra extended performance (XP) was replaced due to malfunctioning and in four cases the connectors were opened, cleaned and reconnected and the impedances were normal again (Table 3).

Of all revised electrodes, 76% were Medtronic ring electrodes, 8% Medtronic directional electrodes, 16% Abbott directional electrodes and 0% Boston Scientific electrodes. The Chi-square test shows a highly significant value. The Chi-square contributions show a deviation for Medtronic ring electrodes and for Boston Scientific electrodes (Table 4).

**Figure 6:**
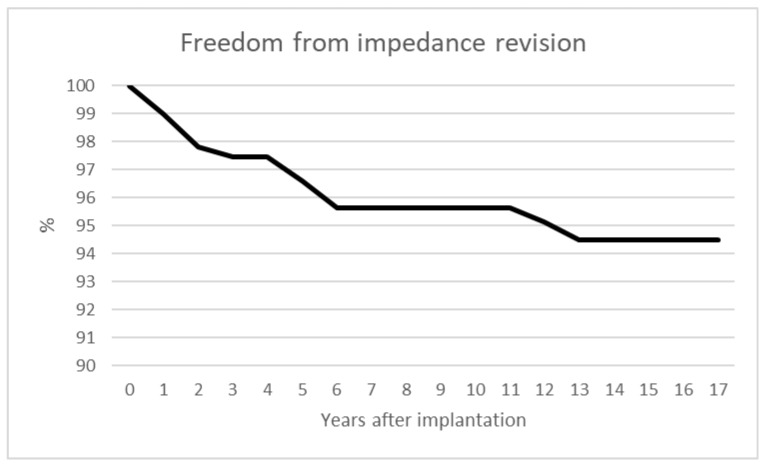
The Kaplan–Meier curve shows freedom from revision surgery due to impedance problems. A total of 52% of revisions were performed within the first two years after implantation; 92% were performed within six years after implantation and 94.5% of systems ran without problems for more than 13 years after implantation.

We analyzed the time intervals of revision surgeries after the de novo implantation to analyze when impedance problems occurred. Six incidents occurred within the first year after implantation, and a further seven within the second year. Two incidents occurred in the 3rd year, four in the 5th year, four in the 6th year and one each in the 12th and 13th year after implantation. A total of 52% of revisions occurred within two years after implantation; 92% of revisions occurred within six years after implantation. For visualization, the data is shown in a Kaplan–Meier curve showing freedom from impedance revision (Figure 6).

#### 3.2.3. Permanent Impedance Problems

Permanent impedance abnormalities occurred in 43 patients either during follow-up or when patients became symptomatic, and reprogramming of the affected contact was possible with satisfying symptom suppression. Permanent abnormalities were observed in 8% of Medtronic systems, 11.2% of Abbott systems, and 3.8% of Boston Scientific systems. Of all systems with permanent impedance abnormalities, 51.16% were Medtronic systems, 30.23% Abbott systems and 18.60% Boston Scientific systems. For Medtronic systems, the expected values for ring contacts is a bit higher than expected; for SenSight systems, the expected values were a bit lower than expected (Table 5).

**Figure 7:**
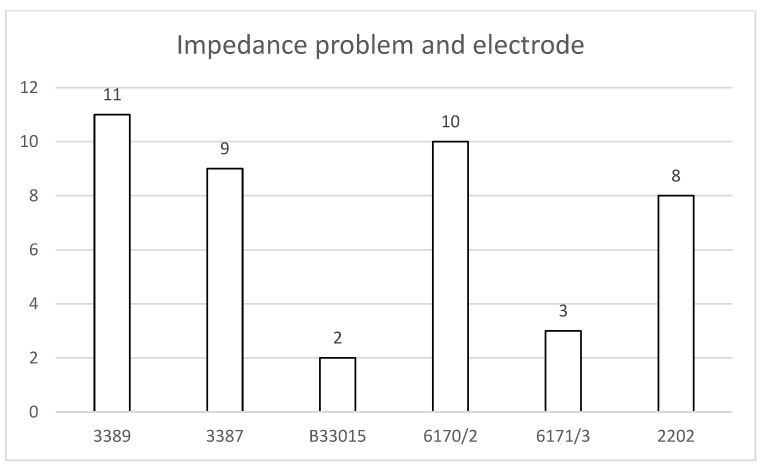
Overview of permanent impedance problems and the implanted electrodes. For Medtronic, short and long spacing are equally affected. For Abbott, the short spacing was affected in the ratio of 3:1.

Because electrodes were most frequently the cause in revised patients, we examined which models were involved. For Medtronic systems, 11 electrodes had narrow spacing and 11 had wide spacing (9 model 3387 and 2 B33015 systems). For Abbott systems, 10 electrodes had narrow spacing and 3 had wide spacing. All eight patients with Boston Scientific systems had narrow-spacing electrodes (Figure 7).

#### 3.2.4. Temporary Impedance Problems

Temporary impedance abnormalities occurred and were resolved in 16 patients during follow-up. These affected 4% of Medtronic systems, 3.4% of Abbott systems, and 0.5% of Boston Scientific systems. Of all systems with temporary impedance problems, 62.5% were Medtronic systems, 25% Abbott systems and 6.25% Boston Scientific systems. The Chi-square test shows a significant value (see Table 6).

**Figure 8:**
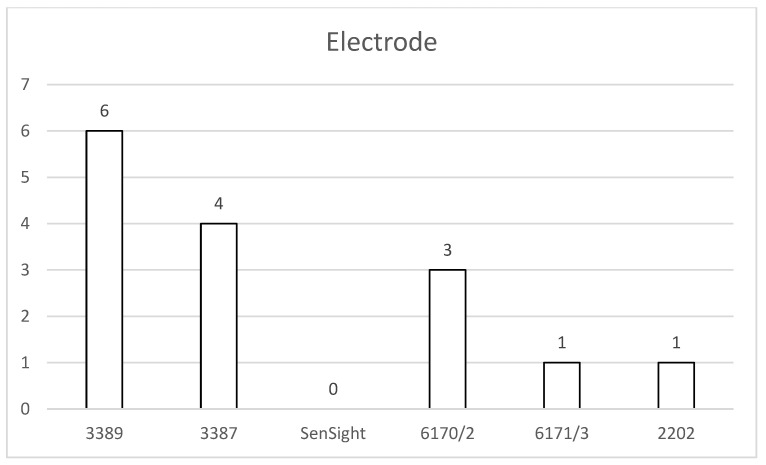
Overview of temporary impedance problems and the implanted electrode. Electrodes with short spacing were affected twice as much.

Again focusing on the electrode models involved, six Medtronic patients had narrow-spacing electrodes and four had wide-spacing electrodes (four model 3387 and zero B33015 systems). Among Abbott patients, three had narrow-spacing electrodes and one had wide spacing. The single affected Boston Scientific patient had a narrow-spacing electrode (Figure 8).

### 3.3. Summary

Overall, 4.16% of all implanted patients (25/601) required surgical revision due to impedance abnormalities. In total, 13.6% of patients (83/601) experienced impedance abnormalities of any type, and only one-third of these cases (25/83) required surgical intervention.

Patients with a tremor experienced significantly more impedance problems needing revision surgery than patients with Parkinson’s disease or dystonia; for patients with PD, this was even less than expected. Electrodes were the leading cause of revision (23 replaced electrodes), followed by extensions (11 replaced), connectors (4 connectors cleaned), and one generator. A total of 52% of the revisions occurred within two years after implantation; 92% occurred within six years. Patients with Boston Scientific systems were affected less than expected by impedance abnormalities causing surgical revision, permanent and temporary abnormalities. In Medtronic systems, ring electrodes with wide spacing were affected more than expected, whereas in Abbott systems, narrow-spacing electrodes were more commonly affected. The directional Medtronic electrodes show comparable incidences to Abbott electrodes. For permanent impedance abnormalities, Abbott systems show higher incidences than expected. For temporary impedance abnormalities, Medtronic ring electrodes show higher incidences than expected.

Our hypothesis that all manufacturers have comparable problems with impedance has to be rejected.

## 4. Discussion

Our patient cohort is similar to other cohorts in big European centers regarding ethnic, gender, age and disease distribution. Dr. Lehrke founded the program in Hamm and performed almost all surgeries, and so the surgical procedures are all comparable regarding the surgical technique. While most centers have only one or two manufacturers, we offer all three manufacturers to our patients.

All patients were treated according to the German guidelines for Parkinson’s disease, essential tremor and dystonia and referred by a specialized neurologist. Fezeu et al. showed that PET-diagnostics is a promising and cost-effective diagnostic tool to diagnose Parkinson’s disease in a very early state (Rapid Eye Movement sleep behavioral disorder) in order to treat patients earlier [20].

Patients with Boston Scientific DBS systems were significantly less affected by impedance abnormalities, likely due to differences in impedance-measurement thresholds. Medtronic and St. Jude/Abbott systems have lower cutoff thresholds than Boston Scientific. Older Medtronic electrodes and Abbott systems define abnormal impedance as >3000 Ω, whereas Medtronic SenSight technology uses >5000 Ω and Boston Scientific uses >9999 Ω. After the introduction of Medtronic SenSight directional leads at our center, the complication rate decreased from 8.01% to 5% for Medtronic systems. Since only symptomatic patients for whom reprogramming was not able to suppress the reoccurring clinical symptoms were treated with revision surgery, the results are still comparable since patients with Boston Scientific systems were not symptomatic even if they had higher impedances than conspicuous impedances in symptomatic patients with Medtronic or Abbott systems.

Impedances change over time. Several groups have shown that impedance generally decreases over time, particularly for active contacts [21,22,23,24]. Lungu et al. reported stable impedances during the first week after implantation, followed by an increase during weeks 1–3 and then stable values for the subsequent five months [25]. Olson et al. likewise demonstrated increasing impedances over time, except for the active contact. Consistent with the earlier findings of Butson et al., they also reported that directional contacts exhibit higher impedances than ring contacts [26,27]. Post mortem studies by Evers and Lowery showed altered neurodegeneration and neuroinflammation around stimulated contacts compared with unstimulated ones, which may explain the impedance decreases observed in most studies [28].

A gradual increase in impedance may also indicate infection with biofilm formation around the contacts, as described by Jaggi and Baltuch [29].

Patients with sudden impedance changes are typically those who become symptomatic or receive an alert from their patient controller. Elberson et al. reported a case of a sudden impedance drop caused by a cyst that developed at the target site three months after implantation. After ceasing stimulation and administering corticosteroids, the cyst resolved and impedance returned to baseline values [30]. In transventricular DBS trajectories, it is known that impedances are lower the closer the contact lies to the ventricles [31]. Impedances below 300 Ω most commonly indicate a short circuit. In our cohort, elevated rather than low impedances were the predominant issue; short circuits occurred in only eight patients.

Deeb et al. described a case of high therapeutic impedance despite normal lead-integrity impedance measurements. They demonstrated that applying constant-current stimulation gradually normalized therapeutic impedance [32]. They also reported an annual lead-fracture rate of approximately 5%. In our cohort, 13.97% of patients exhibited impedance abnormalities, but only 4.16% required revision surgery. Interestingly, half of our revisions occurred within the first two years after implantation (52%), and almost all (92%) within six years after implantation. We would have guessed that the longer a system is implanted, the more prone it is to impedance problems. The Kaplan–Meier curve shows that 94.5% of systems run without problems after 13 years.

**Figure A2:**
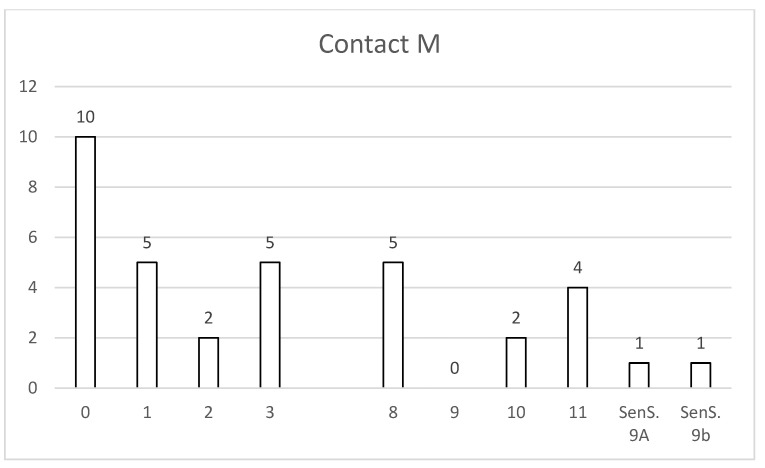
Overview of affected contacts of permanent impedance problems for Medtronic. Contact 0 was most often affected since it was usually the first contact that was screwed. The longer electrode was affected twice as much.

Whitestone et al. also found that electrodes were the primary source of impedance problems (46.1%), consistent with our findings [33]. Fernández et al. reported lead fractures in 4% of electrodes, typically 9–13 mm above the connector [34]. Van der Bouwens Vlis et al. identified wire tethering as the most common cause of impedance abnormalities [35]. Sturgill et al. found that falls were the most frequent cause of revision surgery and that Abbott patients were more affected than Medtronic patients (29.4% vs. 1.7%) [36]. In contrast, the main cause in our cohort was spontaneous impedance abnormalities rather than trauma. More of our Medtronic patients than expected were affected and electrodes with ring contacts and wide spacing showed higher incidences. The older Medtronic extensions used four screw nuts that had to be tightened on each contact; contacts 0 and 9, which were tightened first, were thus most frequently implicated in permanent impedance problems (see Appendix A Figure A2). Here Abbott systems show a higher incidence of permanent impedance problems, which leads to a loss of MRI compatibility.

As shown by Allert et al., activating a neighboring contact often provides adequate symptom control in symptomatic patients with impedance abnormalities. Similarly, only one-third of our patients with impedance abnormalities required revision surgery [37].

In our cohort, 13.98% of patients exhibited impedance abnormalities, consistent with previously reported rates ranging from 2.8% to 55% [8,13,16,36].

Overall, 4.16% of our patients required surgical revision. Electrodes were the primary source of the problem: 6 of 23 affected electrodes were directional leads, and 17 were ring-contact electrodes, suggesting that the Medtronic 3389 and 3387 models were more susceptible. In particular, the 3387 electrodes with wide spacing were twice as often problematic (*n* = 12 vs. *n* = 5 for the 3389). Among directional leads, four Abbott 6170/2 electrodes and two Medtronic B33015 electrodes were affected. Since patients with essential tremor were more likely to be affected by impedance problems that needed surgical treatment, it still remains unclear if the ring electrodes from Medtronic with wide spacing were the cause or if the patients with essential tremor were the cause. Patients with tremor and Abbott systems did not have as many problems. Since the old Medtronic electrodes are no longer manufactured and the SenSight systems do not show more impedance problems than expected, the data suggests that maybe the electrodes were the problem.

The limitations of our study are that we performed an observational and retrospective study and that our patient cohort represents a classical central European patient cohort. So our results may not applicable to centers with a different ethnic and patient population. To gain more detailed insights, a multi-centric, randomized and prospective study design is needed.

To our knowledge, this is the first study to compare impedance abnormalities across all three major DBS manufacturers. A strength of our series is that all procedures were performed by only three surgeons using a highly standardized surgical technique, ensuring strong comparability across cases. We show that the newer Medtronic SenSight electrodes are associated with fewer impedance abnormalities than older models. Patients with tremor were more prone to impedance issues than patients with Parkinson’s disease and dystonia, and wide-spacing electrodes with ring contacts were more frequently affected—a finding that warrants further investigation. Interestingly, in patients with Abbott systems, narrow-spacing electrodes were more often implicated.

## 5. Conclusions

Checking impedances in DBS patients is essential for detecting abnormalities, although most impedance deviations are not clinically relevant. In this study, we demonstrated that directional electrodes are less prone to impedance problems than ring-contact electrodes. Roughly one half of revisions occurred within the first two years after implantation, 92% within six years. Boston Scientific systems exhibited the lowest rate of sudden loss of efficiency and impedance abnormalities. As electrodes were most often the source of the issue, they should be the first component evaluated during troubleshooting. The old Medtronic systems show a higher incidence of temporary impedance problems; Abbott systems show a higher incidence of permanent impedance problems.

## Figures and Tables

**Table 1:** Overview of patients’ characteristics. The majority of patients were Caucasian (97%), with a slight majority of men amongst our patients (59%). The median age was 62 years, ranging from 15 to 85 years. The treated diseases were Parkinson’s disease in more than half our patients, followed by tremor and dystonia.

Population Statistics	
**De novo implantations**	*n* = 601
**Ethnicity**	
Caucasian	583 (97%)
Arabic	15 (2.5%)
Asian	2 (0.3%)
African	1 (0.2%)
**Sex**	
Women	245 (41%)
Men	356 (59%)
**Age**	
Median age	62 (15–85)
**Disease**	
Parkinson’s disease	337 (56%)
Essential Tremor	194 (32%)
Dystonia	70 (12%)

**Table 2:** The Chi-square test for patients who underwent revision surgeries shows a highly significant value of 0.0003. The Chi-square contributions show high values for the revised patients with PD, which is lower than expected, and for patients with ET, which is higher than expected.

Category	Observed Value (O)	Expected Value (E)	Chi-Square Test	Chi-Square Contribution (=(O − E)^2^/E)
PD	36.00	56.07	0.000265397	7.19
ET	48.00	32.28		7.66
D	16.00	11.65		1.63

**Table 3:** Overview of intraoperative findings. Electrodes were the most common cause for impedance problems, followed by extensions and connections, and one St. Jude Libra XP was replaced due to malfunctioning. Medtronic electrodes with wide spacing seem to be more vulnerable; for Abbott electrodes, electrodes with narrow spacing seem to be more vulnerable.

Component/Company	M Ring	M Directional	A	B
Electrode				
-narrow spacing	5	0	4	0
-wide spacing	12	2	0	0
Extension	7	3	1	0
IPG	0	0	1	0
Connection	4	0	0	0

**Table 4:** Overview of revised electrodes due to impedance abnormalities. A total of 76% were Medtronic ring electrodes, 8% were Medtronic directional electrodes and 16% were Abbott electrodes. Chi-square values are highly significant. The Chi-square contribution values show higher values than expected for Medtronic ring electrodes and lower values than expected for Boston Scientific electrodes.

Category	Observed Value (O)	Expected Value (E)	Chi-Square Test	Chi-Square Contribution (=(O − E)^2^/E)
M ring	76.00	39.10	3.179 × 10^−15^	34.82
M directional	8.00	6.66		0.27
A	16.00	19.30		0.56
B	0.00	34.94		34.94

**Table 5:** Of all permanent impedance abnormalities, 51.16% occurred in Medtronic systems, 30.23% in Abbott systems and 18.60% in Boston Scientific systems. The Chi-square test shows a significance of 0.001. The Chi-square contribution values show higher values than expected for Abbott systems and lower values than expected for Boston Scientific systems.

Category	Observed Value (O)	Expected Value (E)	Chi-Square Test	Chi-Square Contribution (=(O − E)^2^/E)
M ring	46.51	39.10	0.001224	1.40
M directional	4.65	6.66		0.60
A	30.23	19.30		6.19
B	18.60	34.94		7.64

**Table 6:** Overview of systems’ temporary impedance problems. A total of 62.5% of affected systems were Medtronic systems, 25% Abbott systems and 6.25% Boston Scientific systems. The Chi-square test is highly significant. The Chi-square contribution values show higher values than expected for old Medtronic systems and lower values than expected for Boston Scientific and Medtronic SenSight systems.

Category	Observed Value (O)	Expected Value (E)	Chi-Square Test	Chi-Square Contribution (=(O − E)^2^/E)
M ring	62.5	39.10	5.95703 × 10^−10^	14.00
M directional	0.00	6.66		6.66
A	25.00	19.30		1.68
B	6.25	34.94		23.56

**Figure A1:**
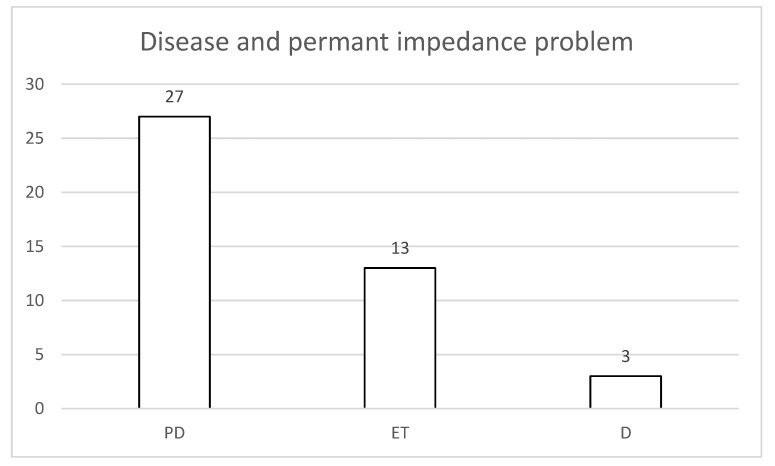
Overview of permanent impedance problems and the disease of the affected patients. The ratio resembles the ratio of diseases implanted in general.

**Figure A3:**
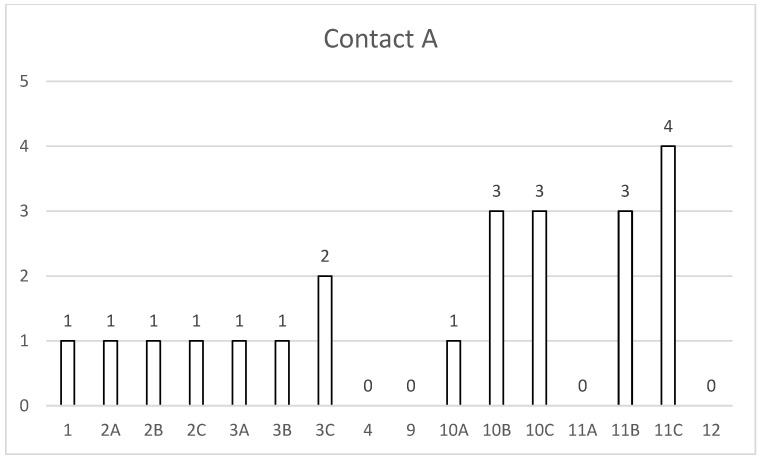
Overview of affected contacts of permanent impedance problems for Abbott. Contacts of Level 3 and 2 were most often affected. The shorter electrode was affected twice as much.

**Figure A4:**
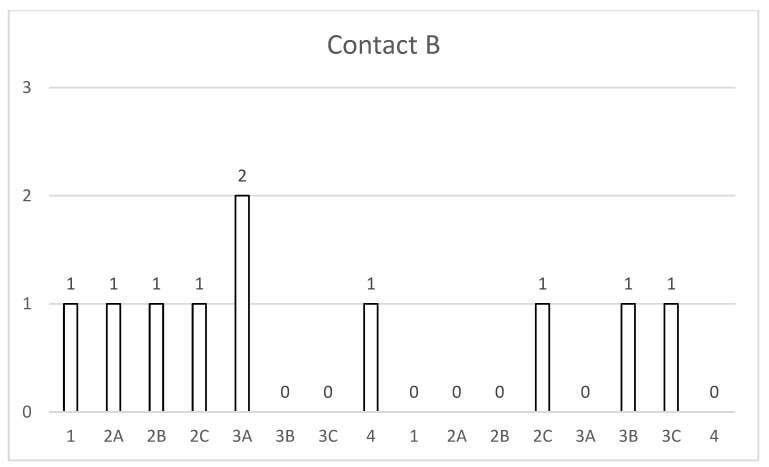
Overview of affected contacts of permanent impedance problems for Boston Scientific. The longer electrode was affected twice as much.

**Figure A5:**
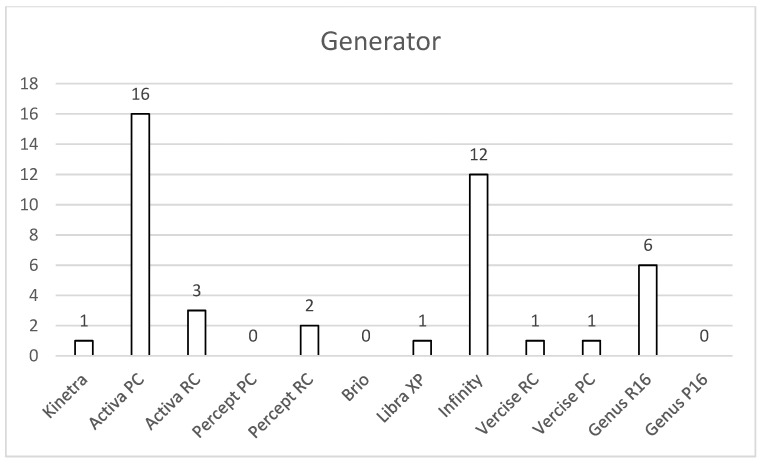
Overview of affected contacts of permanent impedance problems and the implanted generator. The ratio equals the number of generators implanted. PC means primary cell and is a non-rechargeable battery. RC means rechargeable cell. XP stands for extended performance.

**Figure A6:**
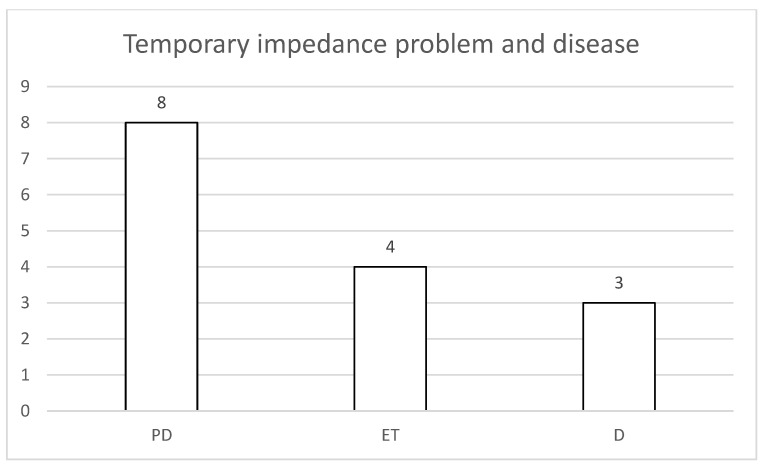
Overview of temporary impedance problems and the disease of the affected patients. The ratio resembles the ratio of diseases implanted in general, except for dystonic patients.

**Figure A7:**
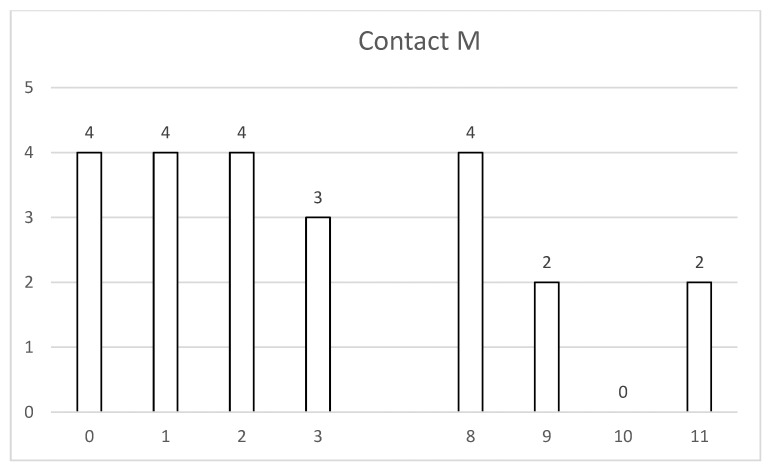
Overview of affected contacts of temporary impedance problems for Medtronic. The longer electrode was affected twice as much.

**Figure A8:**
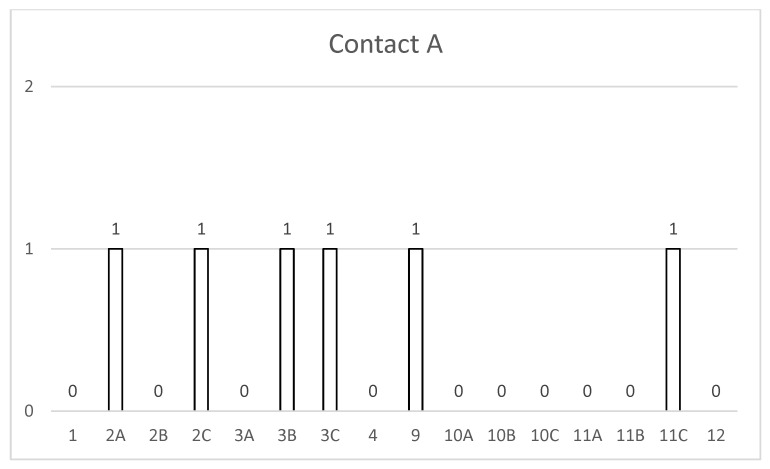
Overview of affected contacts of temporary impedance problems for Abbott. Contacts of Level 3 and 2 were most often affected. The longer electrode was affected twice as much in contrast to permanent impedance problems.

**Figure A9:**
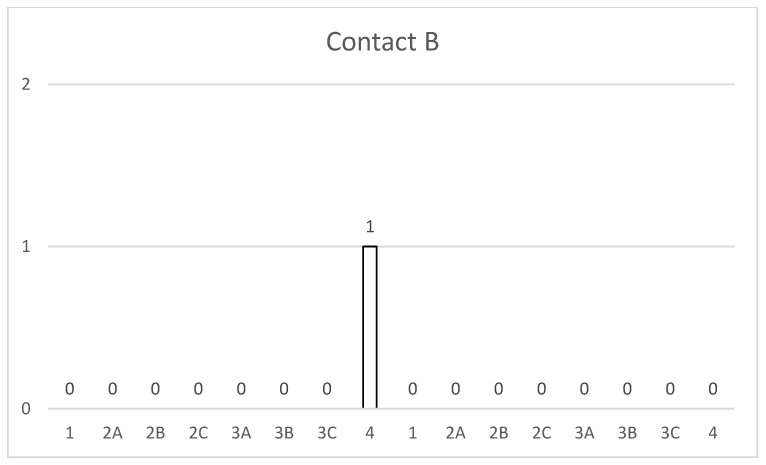
Overview of affected contacts of temporary impedance problems for Boston Scientific.

**Figure A10:**
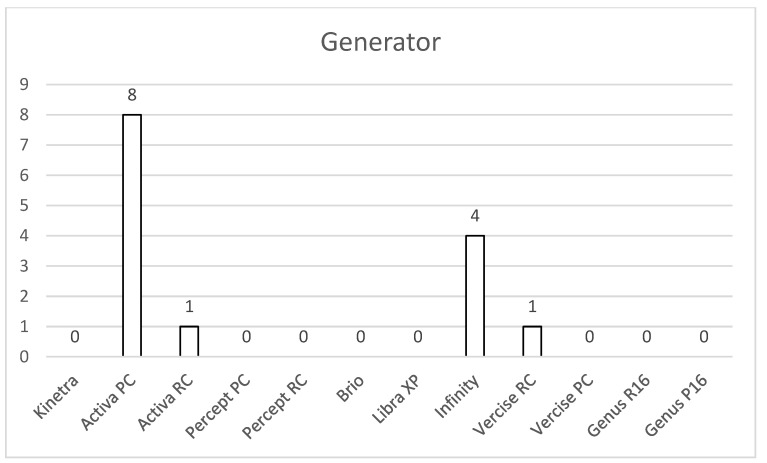
Overview of affected contacts of temporary impedance problems and the implanted generator. The ratio equals the number of generators implanted.

## Data Availability

The original contributions presented in this study are included in the article. Further inquiries can be directed to the corresponding author(s).

## Abbreviations

The following abbreviations are used in this manuscript:

DBS	Deep brain stimulation
ET	Essential tremor
PD	Parkinsons’ disease

## Appendix A

Additional data is presented here of subgroup analyses of permanent and temporal impedance problems.
